# Intervention mechanism of marine-based chito-oligosaccharide on acute liver injury induced by AFB_1_ in rats

**DOI:** 10.1186/s40643-023-00708-6

**Published:** 2024-01-18

**Authors:** Lin Chen, Jiahui Yan, Huijun Shi, Zhaohuan Zhang, YueLiang Zhao, Yong Zhao, Yuan Wang, Jie Ou

**Affiliations:** 1 College of Food Sciences and Technology, Shanghai Ocean University, Shanghai, 201306 China; 2 Engineering Research Center of Modern Preparation Technology of TCM, Shanghai University of Traditional Chinese Medicine, Shanghai, 201203 China; 3 Shanghai Engineering Research Center of Aquatic Product Processing and Preservation, Shanghai, 201306 China; 4 Laboratory of Quality and Safety Risk Assessment for Aquatic Product on Storage and Preservation, Ministry of Agriculture and Rural Affairs, Shanghai, 201306 China

**Keywords:** Aflatoxin B_1_, Chito-oligosaccharide, Oxidative Stress, Apoptosis, RNA-Seq

## Abstract

Aflatoxin B_1_ (AFB_1_) is extremely hepatotoxic, a causative agent of liver cancer, and can cause symptoms of acute or chronic liver damage. Chito-oligosaccharides (COS), obtained from the degradation of chitosan derived from shrimp and crab shells, is a natural antioxidant substance and its antitumor properties have been widely studied, but less research has been done on the prevention of AFB_1_-induced acute liver injury. In this study, rats were acutely exposed to 1 mg/kg BW AFB_1_ and simultaneously gavaged with different doses of COS for 8 days. The results showed that COS attenuated the hepatic histopathological changes and reduced serum biochemical indices (ALT, AST, ALP, and TBIL) in rats. It significantly inhibited MDA content and promoted SOD and GSH-Px activity production. Moreover, it also improved hepatocyte apoptosis. Furthermore, AFB_1_-vs-HCOS differential genes were enriched with 622 GO entries, and 380 were Biological Processes, 170 were Molecular Functions, 72 were Cellular Components. Differentially expressed genes (DEGs) analyzed by KEGG enrichment were more enriched in pathways, such as metabolism, PPAR signaling pathway, and peroxisome. Q-PCR technique verified that *Lama5*, *Egr1*, *Cyp2b1*, and *Gadd45g* in DEGs were associated with oxidative stress damage and apoptosis. In conclusion, COS intervention reduces the effect of AFB_1_ on hepatic genes and thus reduces the changes in hepatic gene function.

**Graphical abstract:**

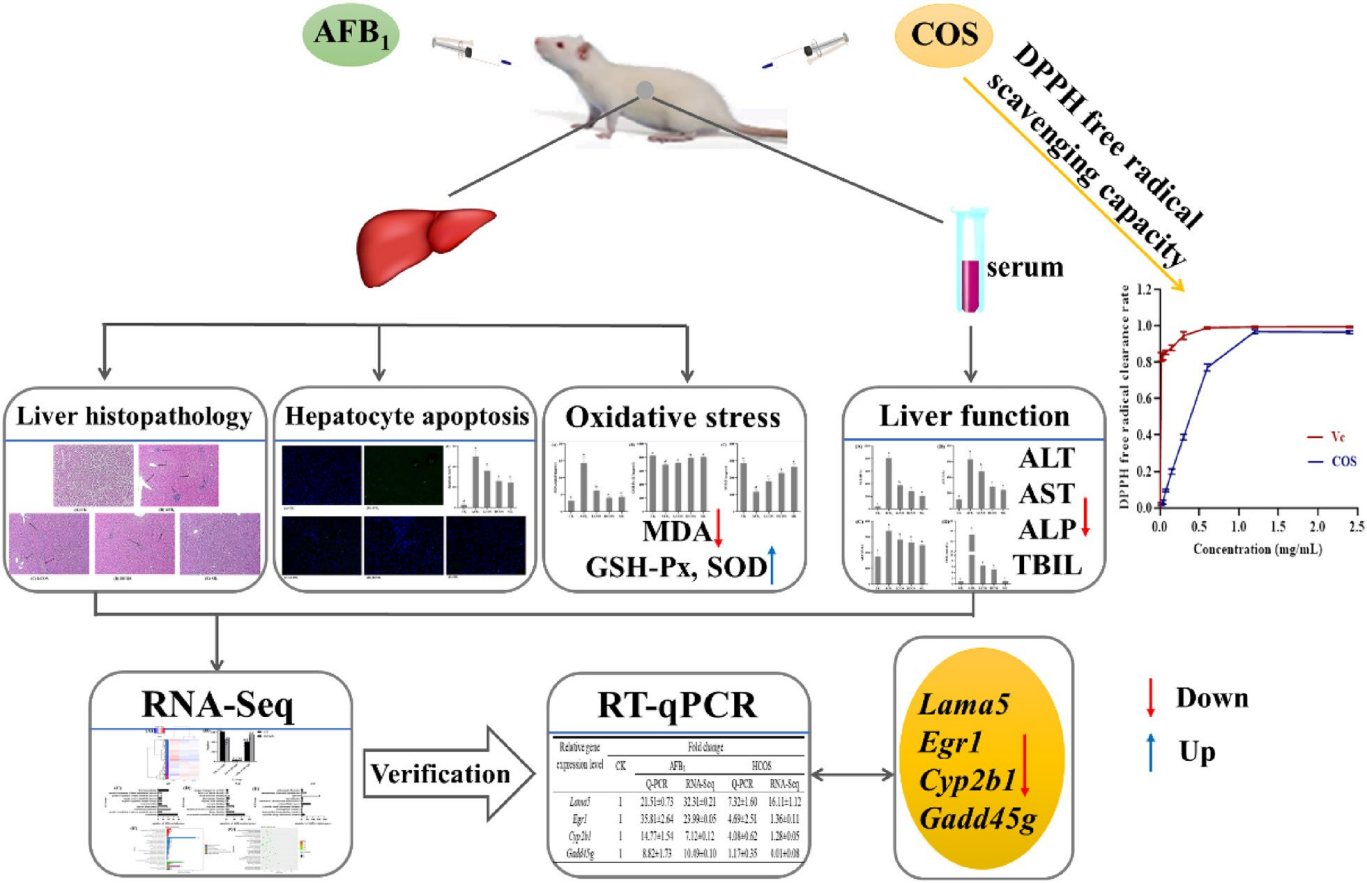

## Key Points

AFB_1_ induced oxidative stress injury and apoptosis of liver.

AFB_1_ activated *Lama5*, *Egr1*, *Cyp2b1*, and *Gadd45g* genes and signaling pathways.

COS attenuated AFB_1_-induced hepatotoxicity.

COS regulated *Lama5*, *Egr1*, *Cyp2b1*, and *Gadd45g* genes and signaling pathways.

## Introduction

Aflatoxins (AFs) are secondary metabolites, which are mainly produced by *Aspergillus flavus* and *Aspergillus parasiticus* (Wu et al. 2009). They are widely present in various food and oilseed crops (grains, oilseeds, nuts, and spices) and even in soil (Conte et al. 2020). Based on its chemical structure, the toxin can be classified into various subtypes, such as AFB_1_, AFB_2_, AFM_1_, AFM_2_, AFG_1_, and AFG_2_ (Limaye et al. 2018). Among them, AFB_1_ is considered to be the most toxic and carcinogenic toxin. It has been found that AFB_1_ can affect the normal functioning of several organs and tissues and has pathogenic effects in epidemiological and animal studies (Xia et al. 2021). Relevant studies have shown that the target organ of AFB_1_ is the liver, and severe damage can cause hepatitis, cirrhosis, and even liver cancer (Hussain et al. 2010). At the same time, different intake times and doses of AFB_1_ can cause acute or chronic liver poisoning. In this paper, the AFB_1_ dose of 1 mg/kg BW was determined on the basis of existing studies, and the equivalent human dose was calculated based on the body surface area conversion factor (Yan et al. 2022; Reagan-Shaw et al. 2008). The result showed that the human intake of AFB_1_ was 0.21 mg/kg, which was lower than the LD50 of AFB_1_. Therefore, the selection of a good method of detoxification has always been a topic of concern.

In animals, AFB_1_ is metabolized into aflatoxin B_1_-8,9-epoxide (AFBO) under the action of microsomal mixed-function oxidase of cytochrome P450 (CYP450) superfamily (Wang et al. 2021). AFBO induces AFB_1_ toxicity production and causes the body to produce large amounts of reactive oxygen species (ROS), which induces oxidative damage in the organism. In addition, AFBO has a high affinity with DNA and can form the adduct AFB_1_-N7-GUA, which leads to DNA mutation and eventually causes hepatocellular carcinogenesis (Woo et al. 2011). AFBO combines with proteins and other macromolecules to form AFB_1_-lysine conjugates, which leads to protein denaturation and even cellular metabolic disorders, apoptosis, and necrosis (Tessari et al. 2010). Therefore, it is crucial to find targets to inhibit AFBO production.

Chitin is a natural polysaccharide widely found in shell of crustaceans, and chitosan and COS products are obtained by deacetylation (Mahata et al. 2014). COS has high value in the development of human nutraceuticals due to its biological properties, such as antitumor, antioxidant, and hypoglycemic (Naveed et al. 2019; Dou et al. 2017; You et al. 2022). At the same time, the intake of COS for its antioxidant properties is known from the literature and is calculated based on the human equivalent dose (HED), which is about 40 mg/kg, and is within the recommended range according to the national regulations on COS as a “new food ingredient” (Reagan-Shaw et al. 2008; Lan et al. 2019). Previous studies have shown that COS has a good ability to scavenge free radicals and increase the activity of antioxidant enzymes in a high-fat diet-induced rat model (Qu and Han 2016). However, the effect of COS on acute AFB_1_-induced liver damage in rats has not been studied. Here, in this study, we investigated the interventional effects of COS on AFB_1_ acutely induced liver damage in vivo by analyzing the changes in phenotypic indicators associated with oxidative stress damage and apoptotic response in hepatocytes.

RNA-Seq sequencing technology is reflecting the expression levels of mRNA, smallRNA, noncodingRNA, etc., or some of the transcripts (Karrer et al. 2021). From the obtained data results, RNA-Seq sequencing technology is often used not only to analyze DEGs, but also to understand the functional signaling pathways of gene enrichment through functional databases. Moreover, the analysis of differential gene functions obtained by RNA-Seq allows to understand the role of external factors or the organism's own effects on gene alterations. By studying the effects of benzo(a)pyrene on zebrafish embryos and larvae, the results showed that DEGs are mainly enriched in disease-related signaling pathways, such as growth failure, organismal death and congenital heart disease (Fang et al. 2015). Therefore, gene sequencing techniques can be used to analyze the functions of DEGs, to understand the causes of external stimuli affecting the organism, and to explore methods of prevention and treatment.

The purpose of this paper is to investigate the intervention effect of COS on AFB_1_-induced acute liver injury in vivo and its mechanism. This study provides a new idea for the prevention and treatment of AFB_1_-induced hepatotoxicity, and also provides a favorable experimental basis for the comprehensive development and utilization of COS.

## Materials and methods

### Chemicals

Aflatoxins B_1_(≥ 99%), dimethyl sulfoxide (DMSO), and silymarin (SIL) were purchased from Shanghai Acmec Biochemical Co., Ltd., Shenggong Biotechnology Co., Ltd., and Tasly Pharmaceutical Co., Ltd. (Shanghai, China), respectively. Chitosan oligosaccharide (≥ 93%, DP 2–20, MW ≤ 1300 kDa), obtained from the degradation of chitosan derived from shrimp and crab shells, was purchased from Shandong Weikang Biomedicine Technology Co., Ltd. (Shandong, China); 1, 1-diphenyl-2-trinitrophenylhydrazine (DPPH) test kit purchased from Beijing Solaibao Technology Co., Ltd. (Beijing, China); The test kits for total RNA extraction were purchased from PowerUp SYBR Green Master Mix (Thermo Fisher Scientific, USA).

### Animals

Fifty 6-week-old healthy male Wistar rats (180–200 g) were purchased from Shanghai SLAC Laboratory Animal Co., Ltd. (Shanghai, China). All rats were housed at room temperature of 22–24 ℃ and a humidity of 50–55%. They ate and drank freely, day and dark alternated for 12 h. All animal experiments were approved by the Living Animal Care and Use Committee for Teaching and Research of Shanghai University of Traditional Chinese Medicine (approval number: PZSHUTCM210115014), and were conducted in accordance with the guidance of the committee.

### Animal experiment

After 1 week of adaptive feeding, 50 rats were divided into 5 groups (*n* = 10). Blank group (CK): 1 mg/kg BW 4% DMSO solution was injected intraperitoneally once on day 6 of normal feeding; Model group (AFB_1_): 1 mg/kg BW AFB_1_ (AFB_1_ dissolved in 4% DMSO solution) was injected intraperitoneally once on day 6 of normal feeding; low-dose COS group (LCOS): 300 mg/kg BW COS by oral gavage once a day, and AFB_1_ once intraperitoneally on day 6; high-dose COS group (HCOS): 600 mg/kg BW COS by oral gavage once a day, and AFB_1_ once intraperitoneally on day 6; Positive drug control group (SIL): 100 mg/kg BW silymarin by oral gavage once a day, and AFB_1_ once intraperitoneally on day 6. Positive drug and the doses were determined based on references (Preetha et al. 2006).

After 8 days, the rats were anesthetized by intraperitoneal injection of chloral hydrate, and the rats were dissected for livers and blood. The liver tissue of the same part of the liver of each rat was soaked in 4% paraformaldehyde solution for histopathological detection, and the remaining liver tissue was stored in the refrigerator at – 80 ℃ for the convenience of subsequent experiments.

### Antioxidant activity of COS

Antioxidant activity of COS was determined using DPPH assay kit. Both COS and vitamin C (Vc) were prepared into solutions of different concentrations (0.01875, 0.0375, 0.075, 0.15, 0.3, 0.6, 1.2, and 2.4 mg/mL). Vc is the positive control. Absorbance was measured at 515 nm.

### Histopathological examination

The liver tissue soaked in 4% paraformaldehyde solution was refrigerated (− 4 ℃) for 24 h, then dehydrated and embedded according to gradient alcohol series, and then used a microtome (RM2016, Shanghai Leica Instrument Co., Ltd., Germany) to prepare the embedded paraffin sections (4 μm). The sections were dewaxed and rehydrated for hematoxylin–eosin (H&E) staining. The histopathological changes of rat liver were observed and photographed under the light microscope (Nikon Eclipse E100, Japan).

### Biochemical analysis

Serum levels of alanine aminotransferase (ALT), aspartate aminotransferase (AST), alkaline phosphatase (ALP), and total bilirubin (TBIL) were measured by automated biochemical analyzer (ADVIA 2120i, Hitachi, Ltd., Japan). The levels of malondialdehyde (MDA), superoxide dismutase (SOD), and glutathione peroxidase (GSH-Px) in liver tissue homogenate were determined according to the detection kit produced by Nanjing Jiancheng Bioengineering Institute (Nanjing, China).

### Hepatocyte apoptosis rate

The paraffin-embedded rat liver sections were dewaxed and repaired with protease K. After the PBS containing 0.1% Triton-X-100 was used to break the membrane, the liver tissue is cleaned and sealed for TUNEL staining reaction, reconstituted with TDT enzyme, re-stained with DAPI, and examined by fluorescence microscope. Under the microscope, the green fluorescent cells are apoptotic cells, and the blue fluorescent cells are living cells. Count the number of hepatocyte apoptosis under light microscope.

### Illumina library generation and RNA sequencing

Total RNA detection kit was used to extract RNA from rat liver tissue. G2965A Agilent 2200 Biological analyzer (Agilent Technologies, Palo Alto, CA, USA) detects the total RNA of samples. Three parallel samples were designed, and the Illumina RNA sequencing method and biological information data analysis was conducted in Azenta.

### Real-time quantitative PCR analysis

Total RNA was extracted from liver samples using TRIzol reagent (Invitrogen, Waltham, MA, USA). NanoDrop (Thermo Fisher Scientific Inc.) was used to determine RNA concentration and purity. The cDNA was synthesized by reverse transcription followed by real-time quantitative PCR sample detection. Primers were designed by randomly selecting 4 genes with *β-Actin* as the internal reference gene (Table 1), and relative expression was calculated according to the 2^−ΔΔct^ relative quantification formula.

**Table 1:** Primer sequences for RT-qPCR

List of genes	Genome ID	Forward primer (5′ → 3′)	Reverse primer (5′ ← 3′)
*Lama5*	ENSRNOG00000053691	ACCTGTGACCCTACAACTGG	GATGACAAGAGCCAGCTTCG
*Egr1*	ENSRNOG00000019422	TTGCCTGTGACATTTGTGGG	GGAAGAGAGGGAAGAGGCAG
*Cyp2b1*	ENSRNOG00000063855	ATACTTTCCTGGTGCCCACA	TCTCCATGCGCAGAAGGTAA
*Gadd45g*	ENSRNOG00000013090	AGCATTGCACGAACTTCTGC	AGCACGCAAAAGGTCACATT

### Statistical analysis

All experimental data were analyzed by GraphPad Prism 8.0 (GraphPad Software, La Jolla, California) and SPSS 26 (IBM, New York, NY, USA). Statistical differences in rat body weight, liver index, biochemical indices (ALT, AST, ALP, and TBIL), oxidative stress index (SOD, MDA, and GSH-Px), and hepatocyte apoptosis rate were calculated using one-way ANOVA, and the Duncan’s test was used for post hoc analysis. The results were expressed by mean ± standard deviation ($\overline{x}$ ± SD). *P* < 0.05 was considered as significant difference.

## Results

### Determination of the ability of COS to scavenge DPPH free radicals

**Fig. 1:**
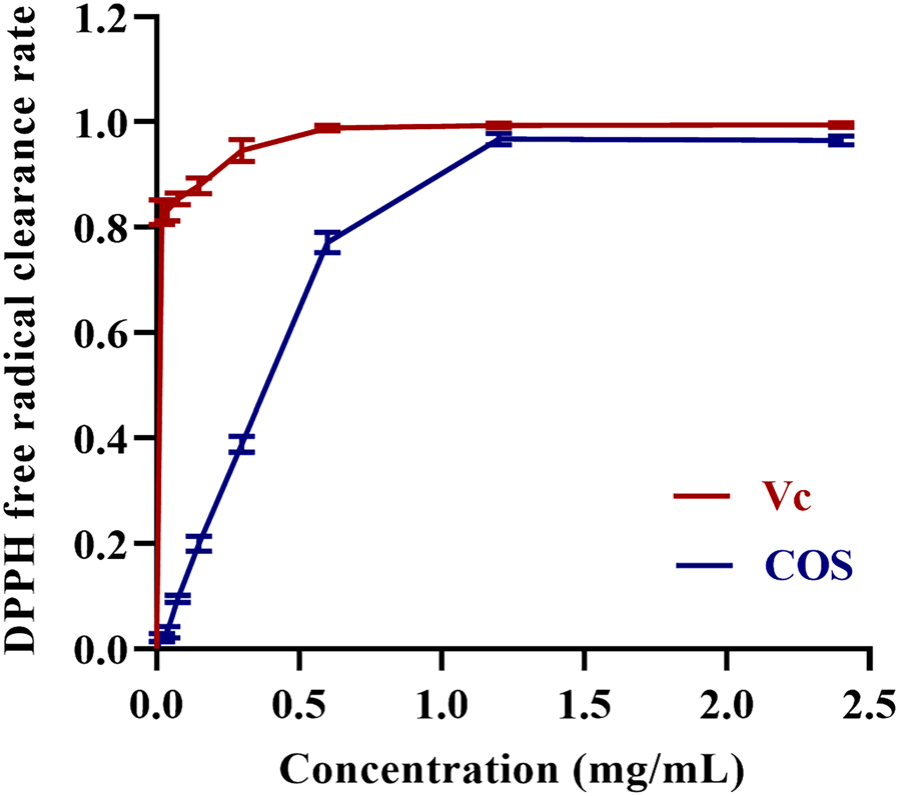
Scavenging abilities of COS and vitamin C on DPPH free radical. *n* = 3. Data are expressed as mean ± standard deviation

DPPH free radical has been widely used to determine in vitro antioxidant capacity due to its hydrogen-donating capacity. Figure 1 shows the results of the scavenging ability of COS and Vc on DPPH free radicals. The DPPH free radical scavenging ability of COS group was dose-dependent with the increase of COS concentration. In particular, the free radical scavenging rate of DPPH reached 96% at concentrations higher than 1.0 mg/mL, which was only close to that of the Vc group. This indicates that COS has good antioxidant properties.

### Effect of COS on body weight and liver index in rats with acute exposure to AFB_1_

**Fig. 2:**
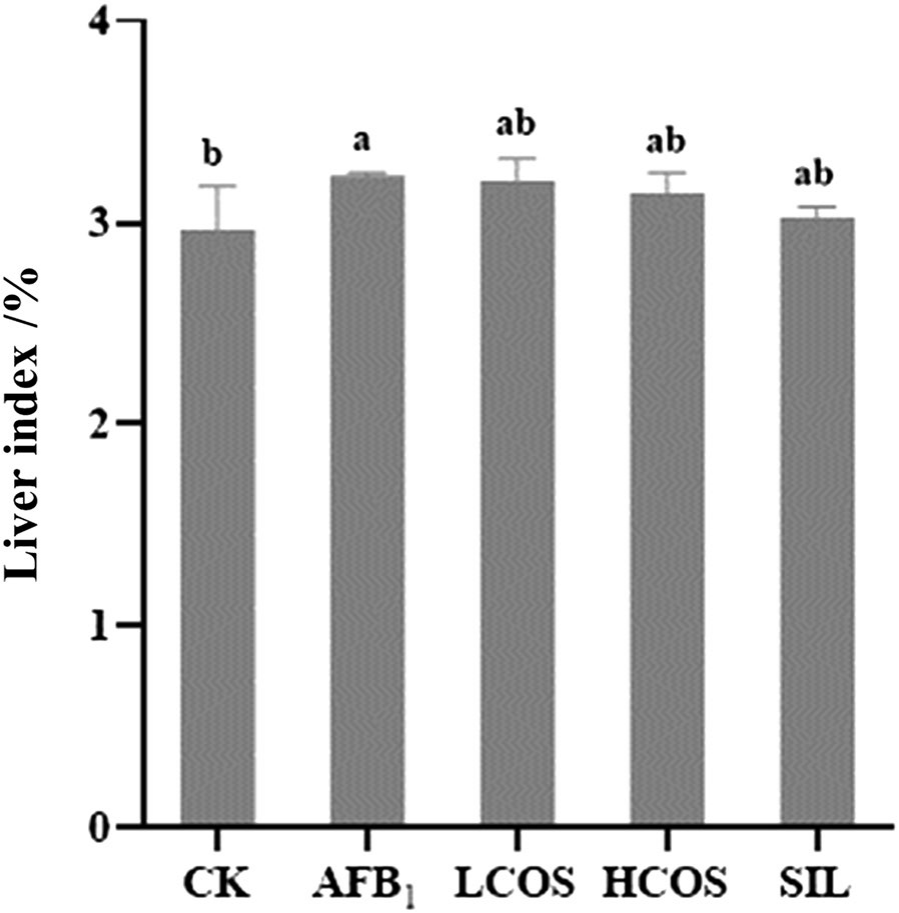
Effect of COS on liver index in rats. *n* = 10. Data are expressed as mean ± standard deviation. Values followed by different superscript lowercase letters (a, b) within the same row are significantly different (*P* < 0.05) according to Duncan’s test

The body weights of rats before and after modeling were recorded during the experiment (Table 2). After 48 h of modeling, the body weight of rats in AFB_1_ group was lower than that in CK group, and the difference was significant (*P* < 0.001). Compared with AFB_1_ group, the average body weight gain of rats in HCOS group and SIL group was significantly increased (*P* < 0.05). As understood from the fluctuation of body weight data, the AFB_1_ group rats showed a negative trend in body weight gain. COS could mitigate the changes in body weight loss in rats caused by AFB_1_. Moreover, organ index is one of the main indicators that respond to the biological characteristics of animals (Zou et al. 2010). The liver coefficient was significantly higher in the AFB_1_ group compared with the CK group (*P* < 0.05). The COS group could alleviate the hepatomegaly caused by AFB_1_ (Fig. 2).

**Table 2:** Effect of COS on body weight in rats

Group	Dose (mg/kg BW)	Body weight (g)			
		Before the experiment	Molding time	After the experiment	Weight gain
CK	–	193.77 ± 3.79^a^	223.57 ± 2.90^a^	229.43 ± 1.45^a^	5.87 ± 1.60^a^
AFB_1_	1	198.80 ± 1.68^a^	222.37 ± 0.79^a^	215.27 ± 0.76^c^	− 7.10 ± 0.54^c^
LCOS	300	196.53 ± 2.74^a^	221.23 ± 1.07^a^	217.67 ± 0.55^bc^	− 3.57 ± 1.22^b^
HCOS	600	193.93 ± 0.98^a^	223.33 ± 0.62^a^	219.83 ± 0.63^b^	− 3.50 ± 0.12^b^
SIL	100	195.10 ± 1.09^a^	224.23 ± 0.58^a^	221.00 ± 0.25^b^	− 3.23 ± 0.38^b^

*n* = 10. Data are expressed as mean ± standard deviation. Values followed by different superscript lowercase letters (a–c) within the same row are significantly different (*P* < 0.05) according to Duncan’s test

### Effect of COS on liver histopathological changes and histopathological changes in rats with acute exposure to AFB_1_

**Fig. 3:**
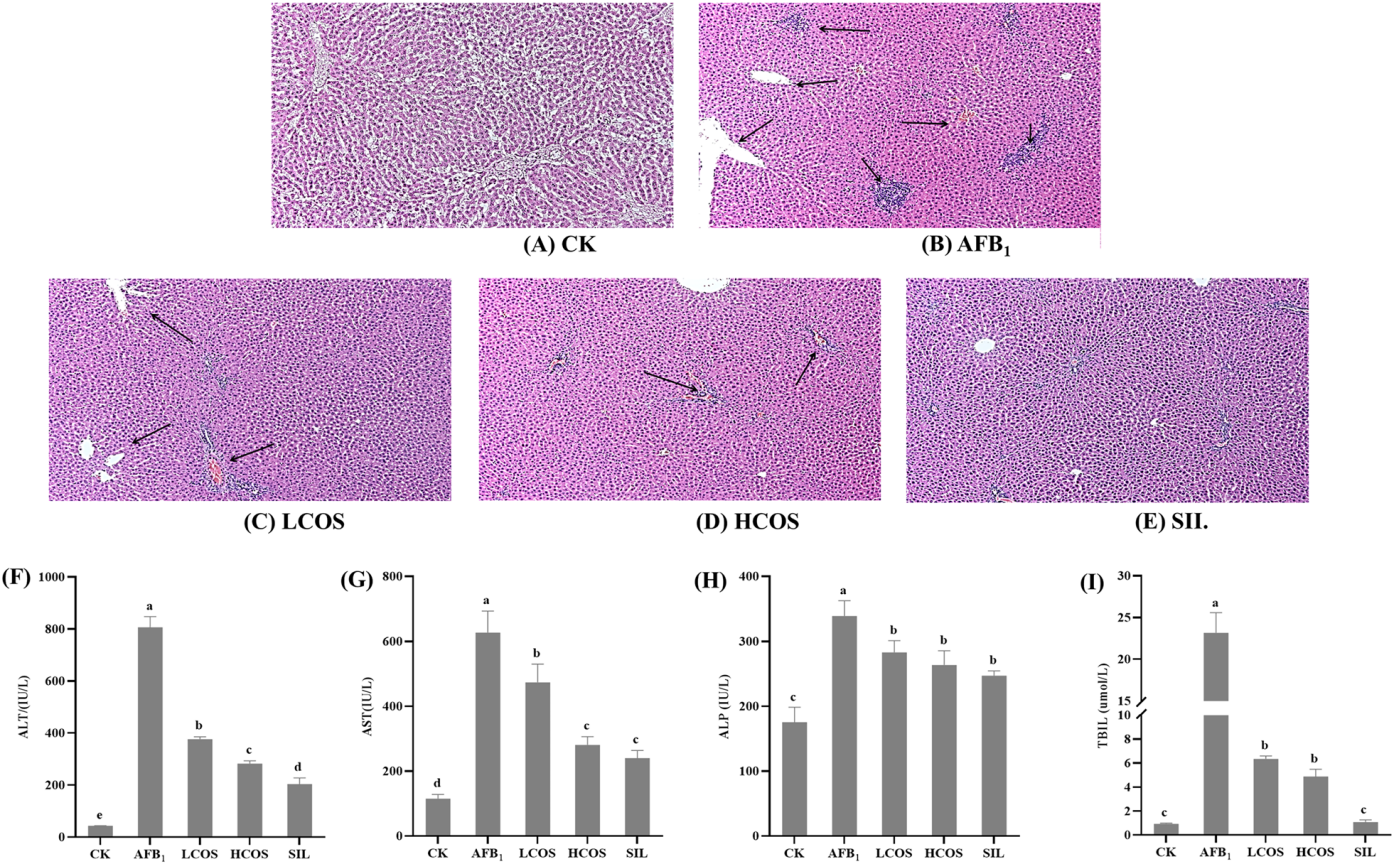
Pathological sections of rat liver tissue. **A**–**E** Representative images of H&E staining (× 100). **A** CK group; **B** AFB_1_ group; **C** LCOS group; **D** HCOS group; **E** SIL group. Effect of COS on serum liver function indicators in rats. **F** Alanine aminotransferase (ALT), **G** aspartate aminotransferase (AST), **H** alkaline phosphatase (ALP) activities, and **I** total bilirubin (TBIL) were measured. *n* = 3. Data are expressed as mean ± standard deviation. Values followed by different superscript lowercase letters (a–d) within the same row are significantly different (*P* < 0.05) according to Duncan’s test

Figure 3A–E visualizes the effect of AFB_1_ on rat liver tissue and the intervention effect of COS. In CK group, the structure of hepatic lobule was complete, and there was no cell necrosis or fibrous tissue hyperplasia (Fig. 3A). In contrast to the normal hepatocyte tissue in the CK group, hepatocytes in the AFB_1_ group were scattered and had small focal necrosis. At the same time, inflammatory cell infiltration with bile duct hyperplasia was observed in the confluent area (Fig. 3B). As seen from the tissue sections, in the intervention group there was decreased liver tissue damage in rats. Among them, only a few hepatocytes necrosis was found in the HCOS group, and mild inflammatory cell infiltration was observed in the LCOS group (Fig. 3C, D). In addition, pathological sections of the SIL group also showed few hepatocytes degeneration and milder inflammatory cell infiltration (Fig. 3E). The degree of liver damage in rats was assessed by measuring serum ALT, AST, ALP, and TBIL activities or levels. The results of liver function index activity of rats in each group are shown in Fig. 3F–I. The levels of ALT, AST, ALP, and TBIL activity were significantly increased in the AFB_1_ group compared with the CK group (*P* < 0.001). This result was consistent with the pathological findings of liver section. Furthermore, the levels of ALT, AST, ALP, and TBIL activity were significantly lower in the intervention group compared with the AFB_1_ group. Meanwhile, the ALT, AST, and ALP activities in the HCOS group were extremely close to those in the SIL group. Therefore, COS can effectively inhibit the dramatic increase of liver function indexes induced by AFB_1_ in rats, and the intervention effect of high-dose COS was relatively better.

### Effect of COS on liver oxidation index in rats with acute exposure to AFB_1_

**Fig. 4:**
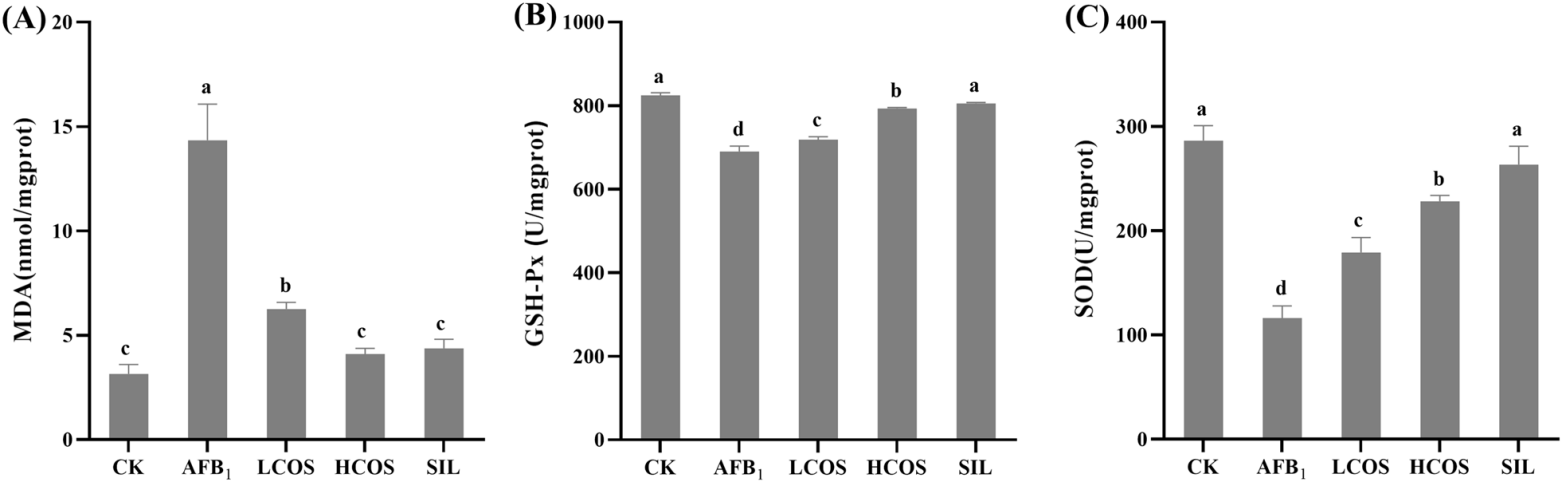
Effect of COS on liver oxidation index. **A** Hepatic malondialdehyde (MDA) content, **B** hepatic glutathione peroxidase (GSH-Px) and **C** hepatic superoxide dismutase (SOD) activity were measured. *n* = 3. Data are expressed as mean ± standard deviation. Values followed by different superscript lowercase letters (a–d) within the same row are significantly different (*P* < 0.05) according to Duncan’s test

MDA content can reflect the degree of lipid peroxidation in the organism. Compared with CK group, the liver MDA content of rats in AFB_1_ group was significantly increased (*P* < 0.001) (Fig. 4A). On the contrary, compared with the AFB_1_ group, the high and low COS intervention groups significantly reduced the liver MDA content of rats. Among them, both the HCOS and SIL groups were able to restore the MDA content to normal levels. According to studies, AFB_1_ was able to induce oxidative stress injury in rat liver tissue. The results of antioxidant enzymes SOD and GSH-Px activities in rat liver are shown in Fig. 4B, C. Compared with CK group, both SOD and GSH-Px activities in rat liver induced by AFB_1_ were significantly decreased (*P* < 0.001). However, compared with AFB_1_ group, the higher COS concentration was, the higher SOD and GSH-Px activities were. It can be seen that the GSH-Px activity in the HCOS group was almost the same as that in the SIL group. Therefore, COS has strong antioxidant activity and scavenges the generation of oxidative free radicals in rats.

### Effect of COS on apoptosis of liver cells in rats with acute exposure to AFB_1_

**Fig. 5:**
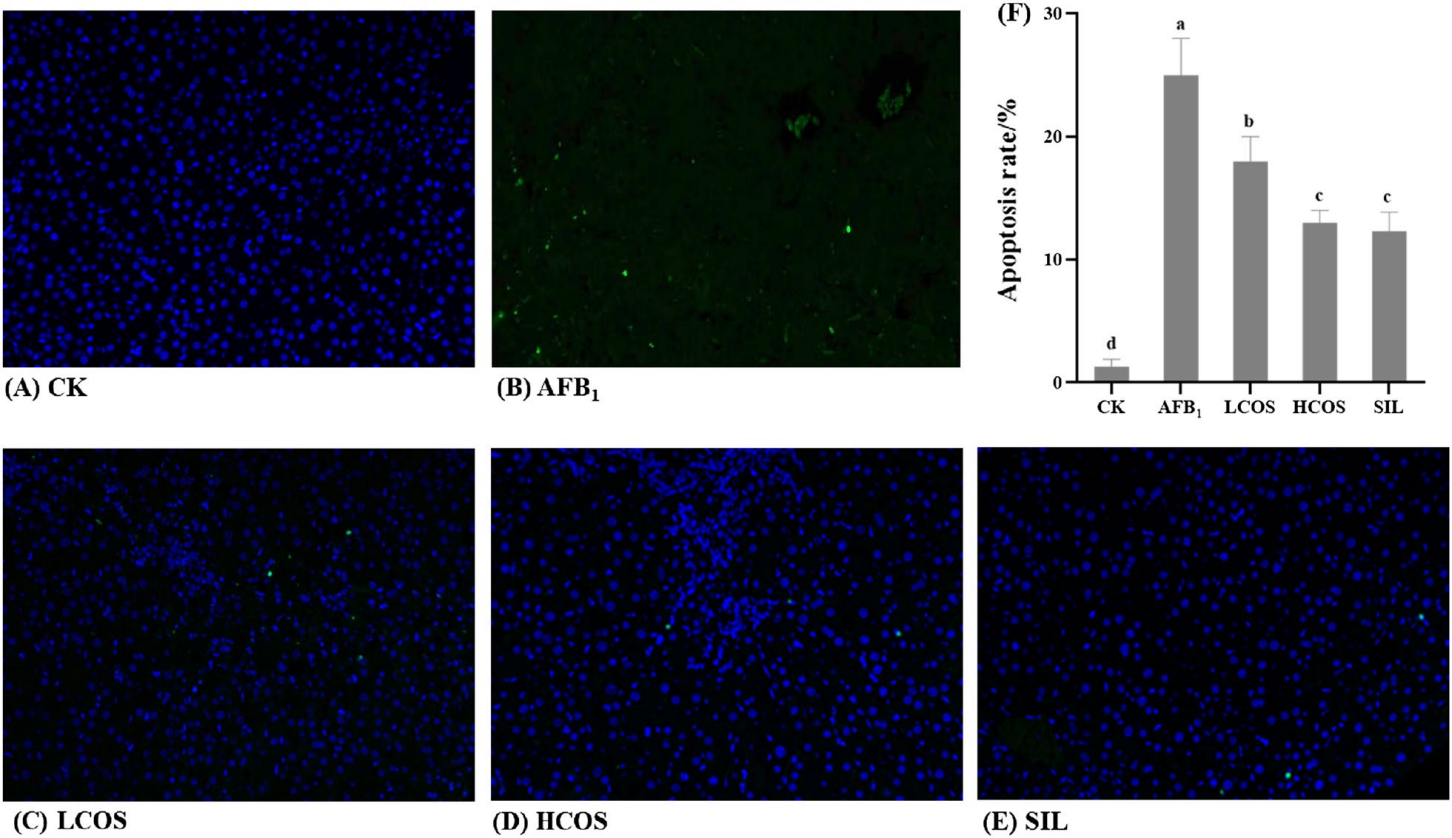
Effects of COS on apoptosis of liver cells in rats. **A**–**E** Representative images of TUNEL staining (× 200); **F** TUNEL assay for percentage of hepatocyte apoptosis. *n* = 3. Data are expressed as mean ± standard deviation. Values followed by different superscript lowercase letters (a–d) within the same row are significantly different (*P* < 0.05) according to Duncan’s test

The apoptosis of rat liver cells is shown in Fig. 5A–F. The hepatocytes in the CK group were stained blue with intact nuclear membranes, and the apoptosis rate was about 1.33% (Fig. 5A). As shown in Fig. 5B, there were green hepatocytes with fragmentary appearance, which indicated serious apoptosis of rat hepatocytes. Nevertheless, the number of green hepatocytes decreased in HCOS group, LCOS group, and SIL group. Moreover, the apoptosis rate was significantly increased in the AFB_1_ group relative to the CK group (*P* < 0.001). However, the apoptosis rate was significantly lower in the high and low COS intervention groups compared to the AFB_1_ group. Among them, the apoptosis rate in the HCOS group was close to that in the SIL group.

### GO enrichment and KEGG pathway analysis

**Fig. 6:**
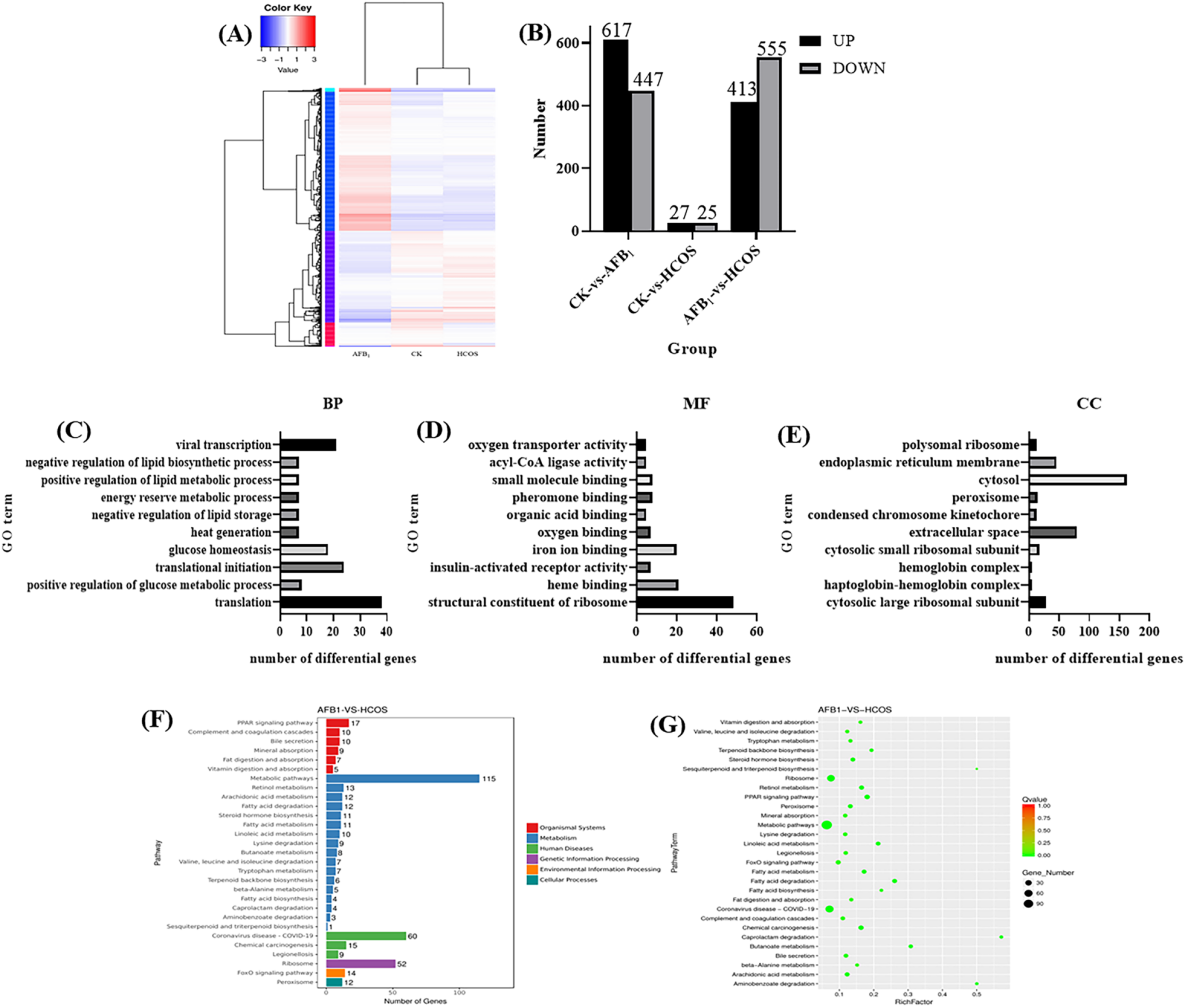
GO enrichment and KEGG pathway analysis of DEGs. **A** Cluster map of differential genes; **B** histogram of up- and down-regulation of differential genes; **C** AFB_1_-vs-HCOS group GO enrichment histogram: BP group; **D** AFB_1_-vs-HCOS group GO enrichment histogram MF group; **E** AFB_1_-vs-HCOS group GO enrichment histogram: CC group. Ordinate: GO term, Abscissa: number of differential genes; **F** annotated classification bar chart of significantly enriched KEGG. Ordinate: pathway, Abscissa: number of differential genes; **G** scatter plot of differential gene KEGG enrichment. Ordinate: pathway term, Abscissa: rich factor, the size of the dot indicates the number of DEGs in the pathway

The study was conducted to understand the similarity of DEGs in CK group, HCOS group, and AFB_1_ group. The experiment took the FPKM value of the differential gene in the sequencing sample as the expression level, and did the hierarchical cluster analysis. Figure 6A shows that the gene expression similarity of the HCOS group was close to that of the CK group. Next, the DEGs were screened and analyzed according to the criteria of FDR ≤ 0.05 and |log_2_FC|≥ 1. Compared with CK group, the AFB_1_ group had 1059 differential genes and HCOS group had 52 differential genes. At the same time, there were 968 different genes in HCOS-vs-AFB_1_ group. Figure 6B shows the up- and down-regulation of the three groups of significant DEGs.

To further analyze the intervention effect of COS on AFB_1_ acute exposure-induced liver damage in vivo at the genetic level, GO enrichment analysis was performed on AFB_1_-vs-HCOS differential genes. 622 GO entries were enriched for AFB_1_-vs-HCOS differential genes. Among them, 380 were Biological Processes (BP), 170 were Molecular Functions (MF) and 72 were Cellular Components (CC). The 10 most significant entries were selected from these three categories separately and the GO enrichment histogram was plotted (Fig. 6C–E). The results showed that the most significant effects were translation (BP), structural constituent of ribosome (MF), and cytosol (CC). The HCOS group mainly had significant effects on the regulation of carbohydrate and lipid metabolism and the initial process of RNA translation, and also altered the effects of acute toxicity of AFB_1_ on heme, oxygen and iron ion binding reactions and oxygen transport activities. Moreover, it is understood that there are deeper effects of AFB_1_ acute toxicity on genes related to the execution of functions on ribosomes, chromosomes and endoplasmic reticulum. In addition, KEGG pathway analysis of differential genes was performed using KEGG database.

The AFB_1_ and HCOS treatment group differentially enriched the 334 KEGG signaling pathways. Table S1 shows the most significant signaling pathways with DEGs were 74 (*P* < 0.05), and the top 29 most significantly enriched pathways were selected (Fig. 6F, G). DEGs of the KEGG pathways were more enriched in metabolism and organismal systems-related pathways. Among them, the metabolic pathway was significantly enriched in DEGs affecting changes in amino acid, carbohydrate, and lipid metabolism in the organism. DEGs in the organismal system pathways were found to be mainly enriched in the PPAR signaling pathway, and the intake of AFB_1_ and COS affected the endocrine, digestive and immune systems of the organism. Besides, DEGs were also mainly concentrated in peroxisomes. Peroxisomes are related to lipid metabolism and cell oxidation level, capable of inactivating toxic substances and oxidizing fatty acids (Argyriou et al. 2016; Kim 2020). Analysis of environmental and genetic information processing-related pathways indicated that the DEGs were mainly concentrated in the FoxO signaling pathway, which acts as a key factor in suppressing carcinogenesis to inhibit cell proliferation and differentiation, oxidative stress, and senescence (Farhan et al. 2017; Lee and Dong 2017).

### Quantitative validation

The *Lama5*, *Egr1*, *Cyp2b1*, and *Gadd45g* genes were highly expressed in DEGs. Quantitative validation of the predicted differential genes based on transcriptome sequencing data was performed for the above genes. Table 3 shows the validation results. The transcriptome sequenced genes using Q-PCR showed the same trend in the AFB_1_-vs-HCOS group.

**Table 3:** Summary of Q-PCR results and RNA-Seq results for randomly selected genes

Relative gene expression level	Fold change				
	CK	AFB_1_		HCOS	
		Q-PCR	RNA-Seq	Q-PCR	RNA-Seq
*Lama5*	1	21.51 ± 0.73	32.31 ± 0.21	7.32 ± 1.60	16.11 ± 1.12
*Egr1*	1	35.81 ± 2.64	23.99 ± 0.05	4.69 ± 2.51	1.36 ± 0.11
*Cyp2b1*	1	14.77 ± 1.54	7.12 ± 0.12	4.08 ± 0.62	1.28 ± 0.05
*Gadd45g*	1	8.82 ± 1.73	10.49 ± 0.10	1.17 ± 0.35	4.01 ± 0.08

*n* = 3. Data are expressed as mean ± standard deviation

## Discussion

AFB_1_ is a mycotoxin that is widely found in moldy foods and cereals and is extremely toxic to humans and animals (Xia et al. 2021). COS obtained from the degradation of marine crustaceans has many important biological activities, including antioxidant and antitumor (Naveed et al. 2019). The aim of this paper was to investigate the interventional effects of COS on single exposure to AFB_1_ in rats and a preliminary investigation of its mechanisms.

The data from this study showed that the antioxidant activity of COS was verified by its free radical scavenging ability on DPPH and this ability was equivalent to vitamin C. This is consistent with previous research results (Yadav et al. 2021). Organ coefficient refers to the percentage of liver weight in whole body weight, which has a certain significance for the evaluation of liver health status. In this study, compared with the AFB_1_ group, HCOS and LCOS group reduced the change in body weight loss induced by AFB_1_. Elevated AST is a response to the degree of damage to hepatocytes, and elevated ALT reflects the activity of liver lesions (Lin et al. 2021). Elevated ALP activity may be associated with diseases such as biliary obstruction, hepatitis, and cirrhosis, and elevated TBIL indicates diminished hepatic metabolic function (Hua et al. 2021). In other words, the elevated AST and ALT activities in the AFB_1_ group (*P* < 0.001) provide a reliable basis for severe liver damage. However, HCOS group showed significant reduction in the activities of AST, ALT, ALP, and TBIL and attenuated the symptoms of AFB_1_-induced hepatotoxicity in rats. Histopathological results showed that only a few inflammatory cells and punctate necrotic cells were found in the HCOS group, thus mitigating AFB_1_-induced liver tissue injury. TUNEL staining results also illustrated that COS intervention reduced the degree of liver swelling, decreased hepatocyte degeneration and necrosis, and inhibited hepatocyte apoptosis. The reduction of apoptosis by COS was similar to the results shown in related studies (Fang et al. 2021). When rats were acutely exposed to AFB_1_, the lipid peroxide MDA content of the liver increased and inhibited the activity of the antioxidant enzymes SOD and GSH-Px. This is due to the increase in MDA which causes changes in the fluidity and permeability of cell membranes. This increase indirectly affects the degree of oxidation and cell damage in the organism (Ahn et al. 2020; Niki et al. 2005). The intervention by COS effectively reduced the level of MDA, as well as increased the activity of antioxidant enzymes (SOD and GSH-Px). This may be related to the antioxidant activity capacity of COS (Karadeniz et al. 2010). Other studies on the biological activities of COS need to be further explored through relevant experiments.

RNA-Seq technique is the most efficient way to obtain unknown genes and signaling pathways in samples. In this paper, DEGs were significantly enriched in metabolism, PPAR signaling pathway, and peroxisome, respectively. The toxicity of AFB_1_ to the liver depends on its metabolic pathway (Lu et al. 2013). AFB_1_ is metabolized by the hepatic phase I metabolic enzyme CYP450 to produce the most detrimental metabolite AFBO, which produces amounts of ROS (Ding et al. 2021). COS acts as a natural antioxidant, reducing the elevated levels of ROS caused by cellular oxidative stress and maintaining the stability of the redox system. This is similar to the experimental results showing that COS increases the activity of antioxidant enzymes as well as enhances the expression of peroxisome pathway (Larasati et al. 2018). Nrf2 is an important antioxidant signaling pathway. It can be found that COS inhibits CYP450 by regulating related metabolic pathways, thus reducing ROS production. Therefore, we hypothesized that the role of COS against ROS is related to the activation of Nrf2 signaling. It was shown that PPAR-γ was shown to enhance the gene expression of antioxidant enzymes (Mohammed et al. 2020). The database predicted that PPAR-γ transcription factors bind to targeted genes (*PEPCK*, *GyK*), and the activator of PPAR-γ upregulates the promoter to promote its expression and produce gluconeogenesis. In addition, AFB_1_ causes oxidative stress in the body, which is also accompanied by lipid peroxidation, inflammatory and apoptotic responses. In our study, genes of COS are enriched in the FoxO pathway, which mainly controls the release of *IL-6* and *IL-10*, activates anti-apoptotic factors, such as ERK1/2 and MEK1/2. ERK1/2 is a member of the mitogen-activated protein kinase (MAPK) family and is involved in cell growth, differentiation, proliferation and apoptosis (Pei et al. 2023). Transcriptome data showed that COS inhibited apoptosis and reduced inflammation by up-regulating ERK1/2 and MEK1/2 through FoxO–MAPK. Moreover, COS decreased ROS expression, which further inhibited the activation of NF-κB signal. Therefore, the results of this experimental study predicted that COS could effectively increase the expression of antioxidant pathway, thus specifically activating PPAR signaling pathway, and increase PPAR-γ protein content and regulate FoxO and NF-κB activation to attenuate AFB_1_-induced oxidative stress and cell proliferation to reduce the secretion of pro-inflammatory factors and exert anti-inflammatory activity. However, its potential research mechanism needs to be further explored.

**Fig. 7:**
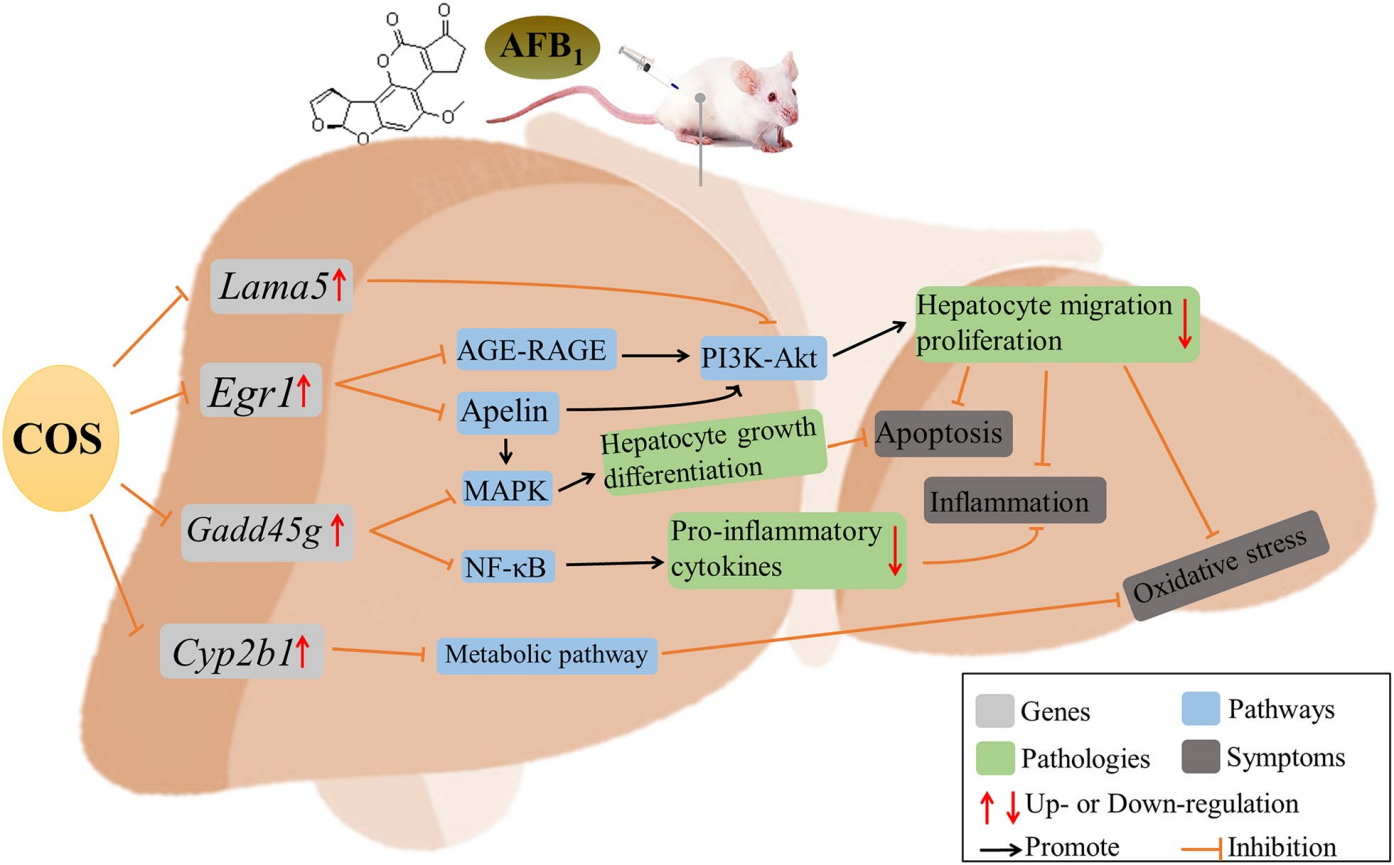
Intervention mechanism of COS on liver injury induced by *Lama5*, *Egr1*, *Cyp2b1*, and *Gadd45g* genes

Four DEGs associated with liver injury were randomly selected by RNA-Seq technique: *Lama5*, *Egr1*, *Cyp2b1*, and *Gadd45g*. Their mechanism of action is shown in Fig. 7. Based on DEGs that were verified by Q-PCR, the *Lama5* gene triggers oxidative stress by specifically activating the PI3K–Akt pathway and promoting hepatic tissue cell migration, proliferation, and vascular endothelial dysfunction (Zhang et al. 2020; Possomato-Vieira et al. 2016). Our results also indicated that *Lama5* was significantly amplified and overexpressed after AFB_1_ exposure. Probably the *Lama5* protein chain is an important component of the extracellular matrix (ECM) and promotes angiogenesis and hepatic metastatic growth. In addition, *Egr1* induces and regulates the expression of multiple genes linked to metabolism, cell division, and tumorigenesis during liver injury (Zhang et al. 2021). In this study, the data of Q-PCR and RNA-Seq were compared. After over-exposure to AFB_1_, the body upregulated *Egr1*, and regulated cell growth and proliferation by regulating p53. COS has a good anti-apoptotic effect, significantly down-regulated *Egr1*, and inhibited cell proliferation and migration. Moreover, we found that *Egr1* can effectively inhibit the occurrence of cancer by mediating Apelin and AGE–RAGE. *Cyp2b1* is enriched in metabolic pathway and chemical carcinogenesis. It has been shown that *Cyp2b1* is involved in retinol production, which decreases liver fibrosis by reducing oxidative stress in the liver, thus suppressing the development of type I collagen and inflammation (Hisamori et al. 2008; Wang et al. 2007). Our data showed a significant decrease in antioxidant enzyme indices and oxidative stress capacity in rat liver after acute exposure to AFB_1_, and intervention by COS alleviated the process of tissue oxidation. This result is similar to that of *Cyp2b1*-mediated Metabolism. Transcriptomic data illustrated that COS intake significantly downregulated *Cyp2b1* and ameliorated oxidative stress injury. It is well-known that *gadd45g* is a growth-arresting, pro-apoptotic protein. The MAPK, NF-κB, FoxO, p53, apoptosis, and cell cycle pathways are all considerably enriched in AFB_1_ and HCOS group. The gene *gadd45g* mediated by NF-κB and p53, which transmit apoptotic signals and reduce pro-inflammatory cytokine expression, prevents the development of tumors (Samivel et al. 2022; Salvador et al. 2013). This is consistent with observations which show AFB_1_ induced hepatocyte apoptosis in rats. The results of control RNA-Seq and Q-PCR studies indicated that AFB_1_ activated *Gadd45g* gene expression and promoted cell growth arrest, which in turn induced acute liver injury in rats. Moreover, both RNA-Seq and Q-PCR results showed that *Gadd45g* was significantly down-regulated by HCOS intervention. The intervention of HCOS made *Gadd45g* mediate the downstream anti-apoptotic gene of MAPK and reduced the apoptosis of body cells. In conclusion, the relative expression levels of *Lama5*, *Egr1*, *Cyp2b1*, and *Gadd45g* verified by Q-PCR were consistent with the expression levels of RNA-Seq results, and the results of HCOS group were more similar to those of CK group.

## Conclusions

In summary, this study initially investigated the intervention mechanism of COS on AFB_1_-induced acute liver injury in vivo. According to RNA-Seq analysis, COS effectively reduces the degree of oxidative stress and the number of apoptotic cells by regulating related signaling pathways and DEGs, thereby inhibiting cell proliferation and differentiation, and ultimately reducing the occurrence of liver damage. The DEGs *Lama5*, *Egr1*, *Cyp2b1*, and *Gadd45g* can be potential target genes for COS treatment of AFB_1_-induced liver injury, but their effectiveness needs to be further explored. Therefore, COS plays a significant role in the prevention and treatment of AFB_1_-induced liver injury.

## Data Availability

All data generated or analysed during this study are included in this published article [and its Additional files], further inquiries can be directed to the corresponding author/s.

## Abbreviations

AFB_1_: Aflatoxin B_1_
COS: Chito-oligosaccharides
DEGs: Differentially expressed genes;
Afs: Aflatoxins
AFBO: Aflatoxin B_1_-8,9-epoxide
CYP450: Cytochrome P450
ROS: Reactive oxygen species
DMSO: Dimethyl sulfoxide
SIL: Silymarin
DPPH: 1,1-Diphenyl-2-trinitrophenylhydrazine
Vc: Vitamin C
H&E: Hematoxylin–eosin
ALT: Alanine aminotransferase
AST: Aspartate aminotransferase
ALP: Alkaline phosphatase
TBIL: Total bilirubin
MDA: Malondialdehyde
SOD: Superoxide dismutase
GSH-Px: Glutathione peroxidase
